# Smear Layer Removal in the Apical Third of Root Canals by Two Chelating Agents and Laser: A Comparative *in vitro* Study

**Published:** 2014-07-05

**Authors:** Hengameh Ashraf, Mohammad Asnaashari, Soheila Darmiani, Reza Birang

**Affiliations:** a Department of Endodontics, Dental School, Iranian Center for Endodontic Research, Research Institute for Dental Sciences, Shahid Beheshti University of Medical Sciences, Tehran, Iran; b Department of Periodontology and Torabinejad Dental Research Center, Dental, Isfahan University of Medical Sciences, Isfahan, Iran

**Keywords:** EDTA, Er, YAG Laser, Etidronate, Scanning Electron Microscopy, Smear Layer

## Abstract

**Introduction: **Smear layer (SL) is produced as a result of mechanical instrumentation of the canal(s). Despite the controversies regarding its removal, the evidence-based trend has shifted towards removing and eliminating the SL. Different methods have been used to remove the SL and the aim of this *in vitro* study was to evaluate the ability of 17% ethylenediaminetetraacetic acid (EDTA), 18% etidronate and Er: YAG on effective removal of the SL. **Methods and Materials:** Fifty straight single-rooted teeth were divided into three experimental groups (*n=*15) and one control group of five. The canals were instrumented with HERO 642 rotary files up to 30/0.06. In group 1, canals were irradiated with Er: YAG laser; in groups 2 and 3, canals were irrigated with 17% EDTA and 18% etidronate, respectively. In group 4 (control) distilled water was used for canal irrigation. The amount of remaining SL was quantified according to Hulsmann’s method with scanning electron microscopy (SEM). Data was analyzed by the Kruskal-Wallis and Mann-Whitney U tests (*P*<0.05). **Results:** The results showed statistically significant differences in terms of SL removal among the groups (*P*<0.05). The amount of removed SL by EDTA was significantly greater followed by Er: YAG laser and 18% etidronate.** Conclusion:** Within the limitations of this study, EDTA was more effective in removing SL compared to Er: YAG and etidronate.

## Introduction

The success of root canal treatment depends on cleaning and shaping, followed by three-dimensional obturation of the root canal system. The mechanical instrumentation of the root canal produces the amorphous irregular smear layer (SL) containing inorganic debris, organic materials like pulp tissue, odontoblastic processes, necrotic debris, microorganisms and their metabolic byproducts [1]. McComb and Smith were the first investigators who showed the presence of a SL in instrumented root canals [2]. Despite the controversies regarding removal of the SL [3, 4], most clinicians have concluded that its presence contributes to leakage and compromises the seal of the root canal filling. It can also serve as a source of nutrients for microorganisms [5, 6]. In a systematic review by Shahravan *et al. *[7] it was concluded that SL removal improves the fluid-tight seal of the root canal system, as suggested by most authors.

Different methods, irrigating solutions and chelators have been used to remove the SL [8]. Currently, the subsequent use of 17% ethylenediaminetetraacetic acid (EDTA) and sodium hypochlorite (NaOCl) is the recommended regiment and gold standard for removal of the inorganic and organic components of the SL, respectively [9]. Recently lasers have been suggested for SL removal and many studies have shown that after irradiation with erbium: yttrium-aluminum-garnet (Er: YAG) laser, most of the SL on the root canal walls was removed and dentinal tubules were patent [10-12].

Etidronate is a member of the hydroxyethylidene bisphosphonate (HEBP) drug family for prevention of osteoclastic bone resorption in patients suffering from bone diseases such as osteoporosis, Paget`s disease and hypercalcemia associated with malignancies (*i.e.* multiple myeloma and breast/prostate cancer) [13, 14]. Etidronate has been recently suggested as an alternative for other chelators because of fewer adverse effects on dentin structure [16].

**Table1:** Scores for smear layer removal (according to the classification by Hulsmann *et al.*) [15]

**Scores**	**Smear layer**
**1**	No smear layer, dentinal tubules are open
**2**	Small amount of smear layer, some dentinal tubules are open
**3**	Homogenous smear layer covers in the root canal wall, only few dentinal tubules are open
**4**	Complete root canal wall covered by a homogenous smear layer, no open dentinal tubules
**5**	Heavy inhomogeneous smear layer covering the complete root canal wall

Moreover, unlike EDTA, etidronate can even be mixed with NaOCl without interfering with the antimicrobial property of this solution [17, 18]. Zehnder *et al*. [17] was the first investigator who used HEBP for SL removal.

Although several studies have evaluated the effect of different methods and substances on SL removal, there is no study on the efficacy of EDTA, Er: YAG laser and etidronate. So, the purpose of this *in vitro* study was to evaluate the SL removal efficacy of etidronate, EDTA and Er: YAG laser in the apical third of root canals.

## Methods and Materials

***Sample collection:***

Ninety-five freshly extracted human mandibular premolars with straight single roots and closed apices were collected. The teeth were decontaminated by immersion in 5.25% NaOCl for 1 h. After obtaining periapical radiographs, all teeth with external or internal root resorption, calcification, complicated root canal anatomy and previous root canal treatment were excluded.

After preparing the access cavity, presence of the canal was confirmed and patency of the canal was established by inserting a #10 K-file (Dentsply, Maillefer, Ballaigues, Switzerland) until the file tip emerged from the apical foramen. The working length (WL) was calculated by subtracting 1 mm from this length. Any root with laterally placed apical foramen or an apical constriction diameter wider than a #15 K-file was excluded. The remaining 50 teeth were selected to a standardized WL ranging between 18-20 mm. The sample size was determined after a pilot study.

***Root canal instrumentation:***

After coding the teeth, they were instrumented up to #20 K-file to the WL. The canals were then passively instrumented using HERO 642 rotary files (Micro Mega, Besancon, France) and an electric speed/torque controller device (X-SMART, Dentsply, Maillefer, Ballaigues, Switzerland) according to the manufacturer’s instructions in a crown-down manner and with the following sequence: coronal enlargement for all the samples by Gates Glidden drills # 4, 3, 2 (Mani Inc., Shioya-gun, Japan) and then preparation of the canals with rotary instruments 20/0.04, 25/0.04, 30/0.04, 30/0.06.

All the canals were prepared by the same operator. Each rotary instrument was used for preparation of five canals and was applied for 5 sec to the WL. After each rotary file, the canal was rinsed with 1 mL of 1% NaOCl, delivered by 28-guage needles (Max-I-Probe, Dentsply, IL, USA) inserted deeply and passively from coronal to middle third at the end of coronal enlargement. A final rinse with 5 mL of distilled water was used to avoid the development of NaOCl crystals and eliminate the irrigation solution from the canals. During the apical preparation sequence, the needle penetrated within the apical 3 mm. The teeth were randomly divided into 4 groups according to the method used for SL removal.

***Group1:*** Fifteen teeth were irradiated with Er: YAG laser (Fidelis, Fotona. Ljubljana, Slovenia) in short pulse (SP) mode through an optical fiber (diameter 0.3 mm), with a wavelength of 2940 nm with an output power of 1 W, pulse energy of 100 mJ and pulse frequency of 10 Hz. The fiber tip was inserted to the WL parallel to the root canal wall and moved with hand circular motion to coronal part of canal during 40 sec (4 times, 10 sec each time, with 15-sec intervals to prevent temperature rise). The percentage of water and air during working was 80% and 70%, respectively. After laser irradiation, the canals were irrigated with 5 mL of distilled water.

***Group 2:*** Fifteen teeth were irrigated for 2 min with 5 mL of 17% EDTA (Clasept, Nordiska dental, Sweden) followed by 5 mL of 5.25% NaOCl and a final irrigation with 5 mL of distilled water to remove any remnants of irrigants.

***Group 3:*** Fifteen teeth were irrigated for 5 min with 5 mL of 5.25% NaOCl followed by 5 mL of 18% etidronate (pH:10.5) for 5 min. Solution of etidronate with 18% concentration (wt/vol) was prepared using purred chemicals (Sigma Aldrich, St Louis, MO, USA) dissolved in deionized water. The solution was stored at 5^º^ C in an airtight dark container. Then it was removed from the refrigerator and stored for 1 min at room temperature, prior to being used.

***Group 4:*** Five teeth were washed by 5 mL of distilled water for 2 min as a final irrigation solution.

***Root sectioning and SEM evaluation:***

Two mesial and distal grooves were prepared on apical 6 mm of each root by means of a disc (3M ESPE, St. Paul, MN, USA) with no entrance into the canal space. The roots were resected transversely in a mesiodistal direction by a chisel (Hu-Friedy, Chicago, IL, USA). The same procedure was repeated in a buccolingual direction and the roots were split longitudinally, resulting in 30 samples in each experimental group and 10 control samples. The samples were placed in 2% gluteraldehyde for 24 h and then rinsed 3 times with a sodium cacodylate buffered solution (0.1 M, pH=7.2). After incubation in osmium tetroxide for 1 h, the samples were desiccated with ascending concentrations of ethyl alcohol (30-100%), placed in a desiccator for 24 h and mounted on a metallic stub. After coating the samples with 20 μm of gold, the technician who was blind to the samples, prepared the SEM photomicrographs using backscatter mode (XL30, Philips, Holland, 2000×). The images were analyzed by 3 previously calibrated examiners according to the scoring system by Hulsmann *et al.* [15] (Table 1). Scores 1 and 2 represented acceptable debridement while scores 3 to 5 represented unacceptable results. The examiners were blinded to sample grouping. In case of disagreement between the examiners for a particular image, a consensus had to be reached.

**Table2.** Evaluation of smear layer in the study groups

	**Scores**				
	Acceptable		Unacceptable		
**Groups (N)**	1	2	3	4	5
**Er: YAG ** **(15)**	0	53.3	46.7	0	0
**17% EDTA** ** (15)**	33.3	60	6.7	0	0
**18% etidronate (15)**	0	0	6.7	93.3	0
**Disstilled water (5)**	0	0	0	0	100

Finally, the data were analyzed using the Kruskal-Wallis and Mann-Whitney U tests in SPSS 17.0 software (SPSS Inc, Chicago, IL, USA). A kappa statistical analysis was performed to measure the inter- and intra-examiner variability.

## Results

**Figure 1:**
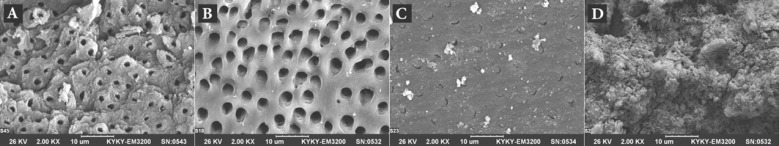
*A*) The apical third of the canal wall after using Er: YAG; small amounts of smear layer is remained, some dentinal tubules are open (score-2); *B)* The canal wall in the apical third, after using 17% EDTA; absence of smear layer, dentinal tubules are open (score-1); *C)* The apical third of the canal wall after using 18% etidronate ; note that the whole root canal wall is covered with a homogenous smear layer, no open dentinal tubules are visible (score-4); *D) *The apical third of the canal wall after using distilled water (control group); heavy inhomogeneous smear layer is covering the root canal wall (score-5)

The kappa values were 0.83 and 0.79, respectively; indicating very good inter- and intra-examiner agreement. Root canal walls absolutely free of SL were not seen in any of the groups (Table 2 and Figure 1). The Kruskal-Wallis test showed statistically significant differences in the amount of remaining SL between the groups (*P*~0.000). The Mann-Whitney U test showed statistically significant differences in the amount of SL between Er: YAG laser and etidronate (*P*~0.000), EDTA and etidronate (*P*~0.000) and between EDTA and Er: YAG (*P*=0.002). EDTA significantly had the best SL removal action followed by Er: YAG laser and etidronate.

## Discussion

This *in vitro* study evaluated the various methods to improve SL removal at the apical third of the instrumented straight root canals. In the current study, the samples in all groups were standardized according to the apical diameter of the root canal. Apical preparation was extended to size 30/0.06 to allow adequate apical penetration of irrigations and access for the laser fiberoptic tip (300 µm) to the apical third of the canals. A similar sequence of HERO 642 files were used in all of the samples for canal instrumentation. It is shown that rotary nickel-titanium (NiTi) files produce a significant amount of SL compared to hand instrumentation [19, 20]. Schafer and Schlingemann reported that debridement of the apical third of the canals was less than the middle and coronal thirds [21]. Arvaniti and Khabbaz also showed that there was a significant difference in presence of SL between apical and middle thirds of the canals [22]. So the removal of SL in the apical region remains unpredictable [23, 24]; therefore we evaluated the apical third of canals.

Injecting the irrigation solution by means of a syringe can control the volume and depth of syringe penetration and results in the flow of the solution to the apical third of the canal [25]. So, all irrigation protocols were done using 28-guage needles, as recommended in other studies [26, 27]. To mimic clinical conditions, all instrumentation and irrigations were done through conventional access cavities. Previous studies have shown that the volume of irrigant and irrigation duration can affect the debridement of the root canal system [28-30]. The protocols used for each method were based on previous similar studies [1, 10, 11, 14].

Different magnifications of SEM have used to score SL after instrumentation. The benefits of this type of study have been reported by some investigators. In accordance with previous studies, a five score index and a magnification of 2000× were used in our study because it offers a detailed image of the canal walls without limiting the observed field.

Certainly, application of laser has shown great promise in root canal treatment and its main usage is to eliminate the microorganisms and remove the remnants of SL on the instrumented root canal walls. Among the different types of lasers, Er: YAG showed the ability to remove the SL [10, 11]. Its wavelength (2940 µm) is absorbed by water and hydroxyapatite. This laser acts through photoablation, so that the water contained in dental hard tissues, evaporates instantaneously and thereby ablates the surrounding tissues with minimal thermal side effects [31]. In current laser system, it is possible to adjust air and water percentage, as two important factors in prevention of temperature damage to attachment apparatus particularly in clinical situations; although our study was done *in vitro*, we adjusted the percentage of water and air at 80% and 70%, respectively. The present study demonstrated that Er: YAG laser can remove the SL but not as effective as EDTA. Similar results were obtained in the study by Ramalho *et al*. [11]. They demonstrated that the optical fiber did not reach all the surfaces of the root canal walls; as a result areas that had a contact with the fiber had no SL. Takeda *et al.* [10, 32] also found that Er: YAG laser can be used to vaporize tissues in the canal and remove the SL. George *et al.* [32] concluded that using radially emitting probes with Er: YAG and erbium, chromium: yttrium-scandium-gallium-garnet (Er, Cr: YSGG) lasers, increased the action of EDTA in removing the thick, artificially created SL. So laser can improve the action of EDTA. Furthermore, several investigators have reported that the effectiveness of laser depends on many factors including the tip to target distance, the power level, the absorption of light in the tissue and the duration of exposure [33-35]. The main purpose for removal of the SL is to eliminate the microorganisms from the root canal and also to disinfect the open dentinal tubules [36]. So, if laser treatment can reduce the number of microorganisms and their by-products and partly open the dentinal tubules, it may yield the identical results of SL removal by other substances like EDTA. Further studies are recommended to evaluate newer generation of radial firing tips that allow lateral emission of the radiation.

The most commonly used irrigation solution in endodontics is NaOCl with well established antibacterial properties and dissolving ability of organic components of tissues [9]; but this solution cannot completely remove the whole SL. Among other chelating substances used in endodontics, 17% EDTA has been superior in removing the inorganic component of the SL [5, 9, 10, 16], which is in agreement with our findings. Nygaard-**Ø**stby was the first investigator who used EDTA to clean the canals [1]. Hasheminia *et al.* [36] also concluded that 1 min application of 17% EDTA was more effective than Er: YAG laser in SL removal.

In this study, the smallest amount of SL removal was observed in 18% etidronate group. This finding confirms that etidronate is a weak chelating agents that had less effect than other commonly used chelators such as EDTA, which is in agreement with previous studies [16, 37, 38]. In our study, we applied etidronate for 5 min, because De-Deus *et al.* [16] showed that this solution needs 300 sec to completely remove the SL. However, the use of this weak chelating agent for longer time periods may potentiate its effect, but this needs more research.

Current preparation methods of using rotary files and irrigation solutions still fall short in successfully removing the SL from the root canal walls. This was confirmed by the results seen in the control group (group 4) where the distilled water was employed as a final irrigation. Although Er: YAG laser has been proven as an effective disinfecting method for the root canal system, the authors recommend using EDTA solution to achieve the optimum results.

## Conclusion

Within the limitations of this *in vitro* study, EDTA showed significantly greater efficacy in removing smear layer followed by Er: YAG laser and finally etidronate.
